# Elevated expression of HIF-lα in actively growing prostate tissues is associated with clinical features of benign prostatic hyperplasia

**DOI:** 10.18632/oncotarget.7641

**Published:** 2016-02-23

**Authors:** Fei Wu, Sentai Ding, Xin Li, Hui Wang, Shuai Liu, Haihu Wu, Dongbin Bi, Kejia Ding, Jiaju Lu

**Affiliations:** ^1^ Department of Urology, Shandong Provincial Hospital Affiliated to Shandong University, Jinan, China; ^2^ Department of Urology, Huashan Hospital, Fudan University, Shanghai, China; ^3^ Department of Radiology, Shandong Provincial Hospital Affiliated to Shandong University, Jinan, China

**Keywords:** benign prostatic hyperplasia(BPH), hypoxia inducible factor-1alpha(HIF-lα), acute urine retention(AUR), fetal prostate(FP), Pathology Section

## Abstract

**Background:**

Benign prostatic hyperplasia (BPH) is one of the most common diseases in middle-age or older men. Increasing evidence has shown that BPH is associated with hypoxia microenvironment.

**Methods:**

We retrospectively collected patient data and tissue samples from fetal prostates(FP), normal prostates(NP), intra-acinar of BPH, peri-acinar of BPH, prostate cancers and sarcomas of prostate. The expression of HIF-1α, as well as VEGF was visualized by immunohistochemistry and statistically analyzed with clinical parameters.

**Results:**

Expression of HIF-lα was observed in intra-acinar of BPH (69.5%), prostate cancer (85.7%) and all FPs, while NP and peri-acinar of BPH tissues were all stained negative. HIF-lα levels in FPs and the malignant tumors were higher than BPH tissues(*p* < 0.05), and the expression of HIF-lα in intra-acinar of BPH was higher than NP and peri-acinar of BPH (*p* < 0.05). The expression of HIF-lα was correlated with the weight of intra-acinar of prostate (*p* < 0.05). And patients with prostate weight larger that 72.45g were prone to have HIF-lα moderate-positive expression, according to the ROC curve (AUC = 0.734, 95%CI = 0.630-0.838). Moreover, the risk of acute urine retention (AUR) for HIF-lα moderate-positive patients increased significantly (OR=5.517, 95%CI = 2.434-12.504).

**Conclusions:**

HIF-lα expression is increased in highly proliferative prostate tissues and correlated with the weight of intra-acinar prostate. Moreover, HIF-lα is also an independent risk factor for AUR occurrence in BPH patients.

## INTRODUCTION

Benign prostatic hyperplasia (BPH) is one of the most common diseases inducing dysuria in middle-age or older men [1]. It is characterized by the lower urinary tract symptoms (LUTS) and bladder outlet obstruction (BOO), and defined by hyperplasia in mesenchyme and glands of prostate histologically. BPH occurs in about one quarter of men in their fifties, half of men in their sixties, and about 83% of all men over 80 years old [2, 3].

Increasing evidence indicates that prostate ischemia is correlated with the pathogenesis of prostate disease [4-6]. Hypoxia-inducible factor (HIF) is a DNA-binding transcription factor responses to hypoxia. HIF-1 is a heterodimer comprising a hypoxic response factor HIF-1α and a constitutively expressed arylhydrocarbon receptor nuclear translocator, namely HIF-1β. HIF-1α binding to transcriptional coactivators can transactivate a specific subset of genes including erythropoietin (EPO), vascular endothelial growth factor (VEGF) [7].

Clinically, high HIF-1α expression is correlated with tumor invasion, lymph node metastasis, distant metastasis and pTNM stage [8]. HIF-1α is increased in prostate cancer and thus drive the transcription of hypoxia-adaptive under both hypoxia and normoxic conditions [9]. In addition, HIF-1α was also present in BPH specimens from patients treated with prostatic surgery. Berger's study indicated that prostatic stromal cells respond to hypoxia by upregulating several growth factors [10]. Moreover, it is well known that VEGF and insulin-like growth factor-1(IGF-1), two crucial downstream effectors of HIF pathway, mediate the pathogenesis of BPH [11, 12].

Recent data from mouse models of spontaneously-hypertensive-rat have shed light into the role of HIF-1α in BPH pathogenesis [6]. To date, however, no clinical evidence regarding the association between HIF-1α and prognosis of BPH has been reported. Therefore, the present study was to evaluate the role of HIF-1α in the pathogenesis and clinical features of BPH.

## RESULTS

### Different expression of HIF-lα in various kinds of prostate tissues

Immunohistochemistry was used to identify the expression of HIF-lα protein in FP, NP, PCa, intra-acinar or peri-acinar of BPH and sarcomas of prostate. HIF-lα protein was mainly distributed in the cytoplasm of malignant tumor cells. In the intra-acinar of prostate from BPH patients, HIF-lα protein was predominantly stained in cytoplasm of epithelia cells.

In all the prostatic tissues above, positive expression of HIF-lα was observed as follows (Table 1): 69.5% in intra-acinar of BPH, out of which moderately and weakly positive were 33.7%(32/95) and 35.8%(34/95) respectively; 85.7%(6/7) in malignant tumors of prostate, with 5 strongly positive and 1 moderately positive, 2 cases of prostatic sarcoma were stained strongly positive (Table 2); all FP were stained positive, 3 strongly positive, 7 moderately positive and 2 weakly positive (Table 3); NP and peri-acinar of BPH tissues were all stained negative.

**Table 1:** Expression of HIF-lα in all prostate tissues

HIF-lαLevels	FP	NP	Peri-acinar	Intra-acinar	Prostate cancer	Prostatic sarcoma
Strongly positive	3/12	0/10	0/12	0/95	5/7	2/2
Moderately positive	7/12	0/10	0/12	32/95	1/7	0/2
Weakly positive	2/12	0/10	0/12	34/95	0/7	0/2
Positive rate (%)	100	0.0	0.0	69.5	85.7	100

FP: fetal prostate; NP: normal prostate

**Table 2:** Expression of HIF-1α in prostate cancer

Gleason score	5	6	7	7	7	8	9
HIF-1α levels	++	﹣	+++	+++	+++	+++	+++

**Table 3:** Expression of HIF-1α in fetal prostate

GA (weeks)	20	21	23	24	24	27	27	28	28	28	30	32
HIF-1α levels	++	+	++	+	++	++	++	+++	+++	++	+++	++

GA: gestational age

**Figure 1:**
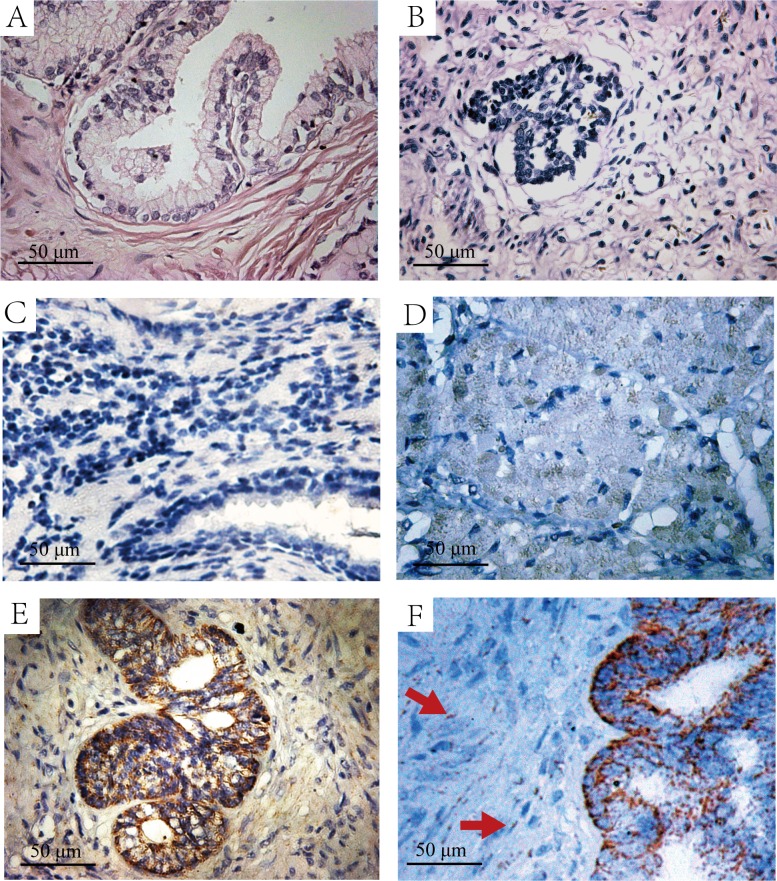
The H&E and immunohistochemistry staining of HIF-lα in normal prostate and fetal prostate tissues **A.** H&E staining of normal prostate tissues. **B.** H&E staining of the fetal prostate tissue. **C.** Immunohistochemistry staining with HIF-lα antibody in normal prostate tissues, which is negative in HIF-lα expression. **D.** Immunohistochemistry staining with HIF-lα antibody in a 21w fetal prostate tissue, the graph shows weak-positive expression. **E.** Immunohistochemistry staining with HIF-lα antibody in a 28w fetal prostate tissue, the graph shows strong-positive expression. **F.** Immunohistochemistry staining with HIF-lα antibody in a 30w fetal prostate tissue with shorted hematoxylin staining time. The graph shows strong-positive expression of HIF-lα, and expression of HIF-lα in mesenchymal cells(Red Arrow).

**Figure 2:**
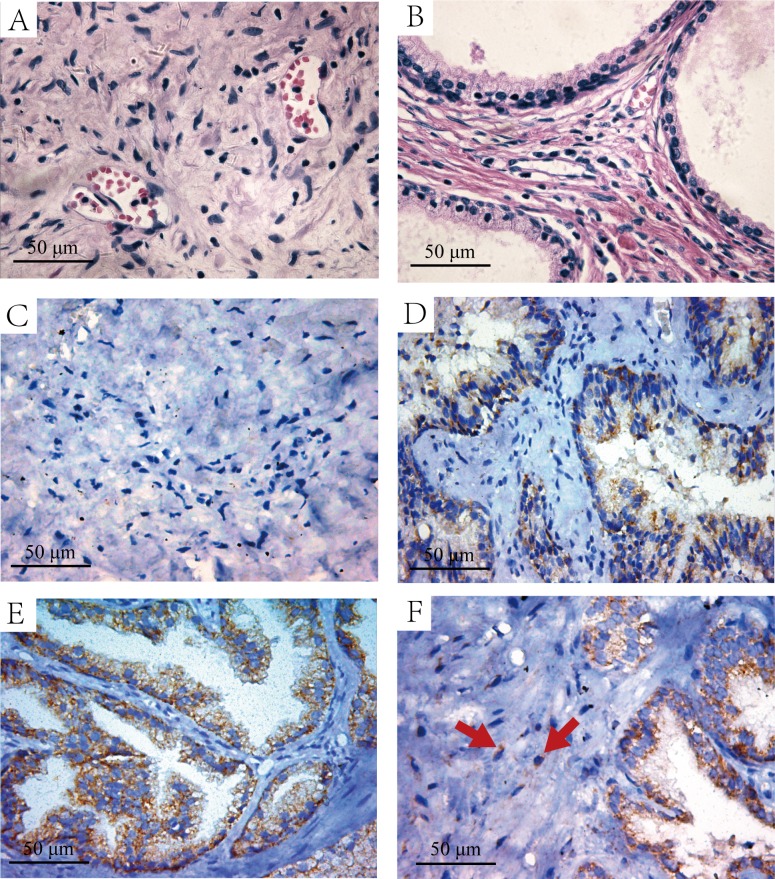
The H&E and immunohistochemistry staining of HIF-lα in the intra-acinar and peri-acinar of BPH tissues **A.** H&E staining of the peri-acinar of BPH tissues. **B.** H&E staining of the intra-acinar of BPH tissues. **C.** Immunohistochemistry staining with HIF-lα antibody in peri-acinar of BPH tissues, which is negative in HIF-lα expression. **D.** Immunohistochemistry staining with HIF-lα antibody in intra-acinar of BPH tissues, the graph shows weak-positive expression. **E.** Immunohistochemistry staining with HIF-lα antibody in intra-acinar of BPH tissues, the graph shows moderate-positive expression. **F.** Immunohistochemistry staining with HIF-lα antibody in intra-acinar of BPH tissues, the graph shows HIF-lα expression in mesenchymal cells (Red Arrow).

**Figure 3:**
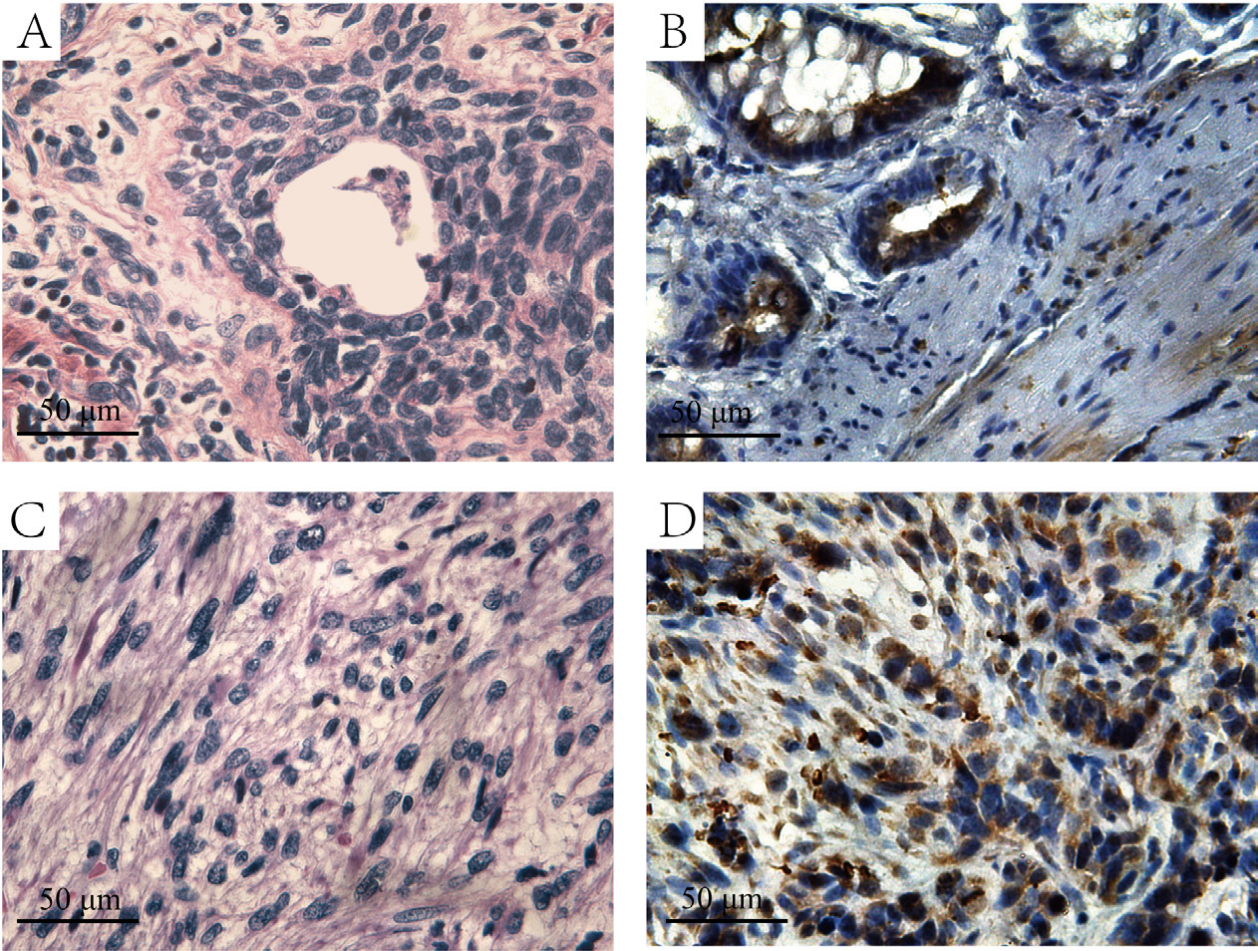
The H&E and immunohistochemistry staining of HIF-lα in prostate cancer and sarcoma tissues **A.** H&E staining of the prostate cancer tissues. **B.** Immunohistochemistry staining with HIF-lα antibody in prostate cancer tissues. **C.** H&E staining of the prostate sarcoma tissues. **D.** Immunohistochemistry staining with HIF-lα antibody in prostate sarcoma tissues, and the graph shows strong-positive HIF-lα expression.

The expression of HIF-lα protein in the tissues of FP and prostatic malignant tumors were higher than BPH tissues (*P* < 0.05), and the expression in intra-acinar of BPH was higher than those in the tissues of NP and peri-acinar of BPH (*P* < 0.05, Figures 1 and 2). Further comparison on the strongly positive rate indicated that malignant tumors (77.8%, 7/9) had a higher incidence rate than FP tissues (25.0%, 3/12), while intra-acinar of BPH was lower than FP tissues (*P* < 0.05, Figure 3). It is noteworthy that HIF-lα was also detected in mesenchymal cells of 7 cases of intra-acinar of BPH (7.4%,7/95, Figure 2F). The staining rate ranged from 5%∼20% according to the counting methods mentioned above. It's in accordance with the standard of positive expression. In addition, HIF-lα expression in mesenchymal cells was also detected in 8 cases of FP tissues (66.7%,8/12, Figure 1F).

**Figure 4:**
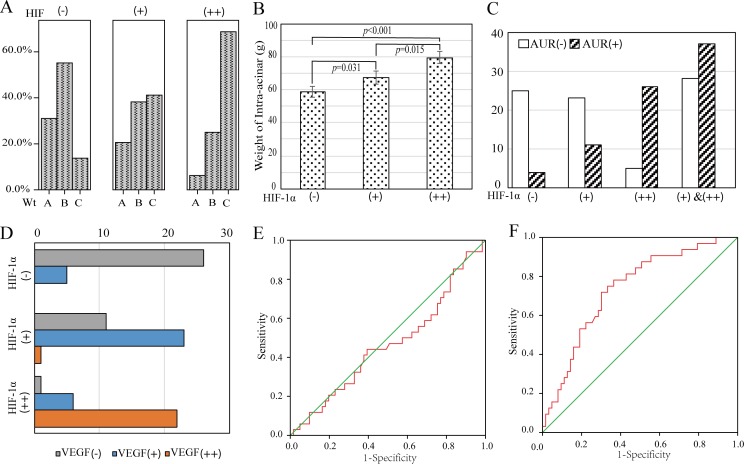
The correlation between HIF-lα expression and clinical variables of BPH patients **A.** BPH cases were classified into three groups according to levels of HIF-lα expression. **B.** The difference of intra-acinar Wt is statistically different when grouped by HIF-lα expression. **C.** The proportion of patients with AUR history is increased with the elevation of HIF-lα expression. **D.** The expression of VEGF in BPH intra-acinar tissues is correlated with HIF-lα. **E.** The receiver operating characteristic(ROC) curve was plotted using weak-positive expressing HIF-lα as status variables and intra-acinar weight of prostate as independent variable. **F.** The ROC curve was plotted using moderate-positive expressing HIF-lα as status variables.

**Figure 5:**
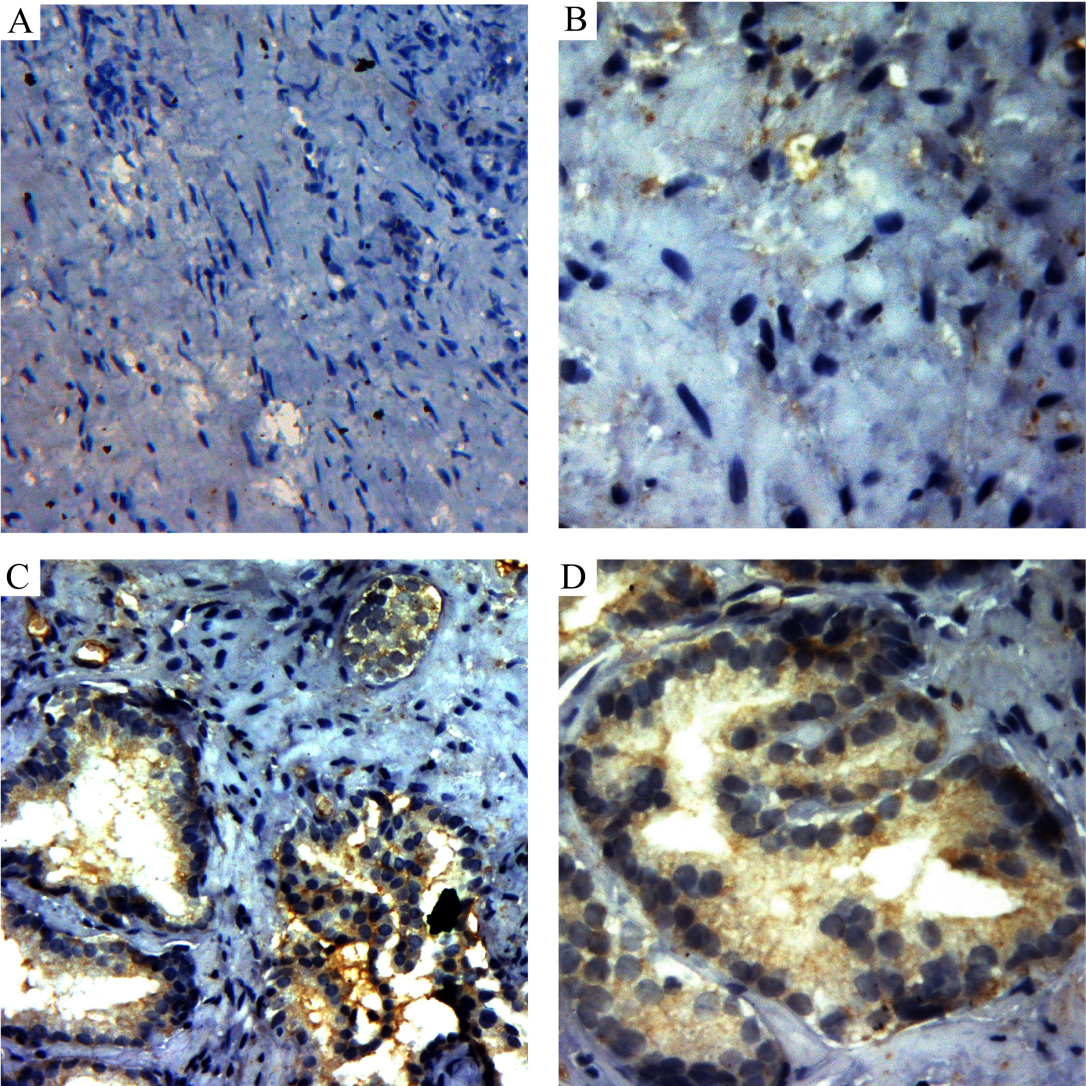
Immunohistochemistry staining of VEGF in the intra-acinar and peri-acinar of BPH tissues **A.** Immunohistochemistry staining with VEGF antibody in peri-acinar of BPH tissues, which is negative in HIF-lα expression(200X). **B.** Immunohistochemistry staining with VEGF antibody in peri-acinar of BPH tissues(400X). **C.** Immunohistochemistry staining with VEGF antibody in intra-acinar of BPH tissues (200X). **D.** Immunohistochemistry staining with VEGF antibody in intra-acinar of BPH tissues(400X).

The expression of VEGF in BPH intra-acinar tissues is correlated with HIF-lα (Figure 4D, Pearson Correlation = 0.799, *P* < 0.01). VEGF cannot be detected in peri-acinar of BPH tissues (Figure 5A & 5B), but is highly expressed in the cytoplasma of intra-acinar cells, as well as extracellular matrix (Figure 5C & 5D).

### HIF-lα protein expression in relation to the weight of intra-acinar of prostate

In Wt groups A,B and C, the rates of weakly positive expression were 38.9%, 34.2% and 35.9% respectively, with moderately positive expression were 11.1%, 23.7% and 53.8% respectively (Table 1). The total positive rates of Wt groups A, B and C were 50.0%, 57.9% and 89.7% respectively. Statistical analysis showed that Wt group C (36/40) had a higher total positive rate than group A (9/18) and B (21/37) (*P* = 0.002 & *P* = 0.001), while there was no significant difference between Wt group A and B (*P*>0.05). When cases were classified according to levels of HIF-lα expression, weight of intra-acinar in HIF-lα negative group was in a normal distribution (Mean = 58.54g, SE = 3.178). While in patients with HIF-lα weak positive group, the mean weight of intra-acinar was 67.31g (SE = 3.69). And the mean weight in moderate positive group was 79.44g (SE = 3.62). ANOVA test indicated that differences among groups and within groups were statistically significant (*P* < 0.01, Figure 4B). To further explore the relationship between intra-acinar weight and HIF-lα expression among 95 BPH patients, receiver operating characteristic(ROC) curve were plotted using HIF-lα as status variables. Intra-acinar weight of prostate failed to sensitively or specifically predict the exist of HIF-lα expression (AUC = 0.464, SE = 0.063,95%CI = 0.341-0.587, Figure 4E). However, moderate positive group has a larger area under cure(AUC) than the weak positive group (AUC = 0.734, SE = 0.053,95%CI = 0.630-0.838, Figure 4F). Moreover, the maximum value of Youden Index (sensitivity = 0.719, specificity = 0.698) is corresponding to 72.45g of intra-acinar prostate weight, which was selected as the cut-off point. In conclusion, the expression of HIF-lα was significantly correlated with the weight growth of intra-acinar of BPH (*P* < 0.05).

### HIF-lα protein expression in relation to incidence rate of AUR

42 cases among all 96 BPH patients suffered AUR, the incidence of AUR in HIF-lα-moderately positive, weakly positive and negative groups were 84.4%(27/32), 32.4%(11/34) and 13.8% (4/28) respectively (Figure 4C). The proportion of patients with AUR history is increased with the elevation of HIF-lα expression (Figure 4C). Compared with HIF-lα negative BPH cases, patients with HIF-lα weak positive did not have a statistically increased risk of having AUR (OR = 1.274, 95%CI = 0.969-1.677). However, the risk of AUR for BPH patients who had moderate-positive HIF-lα expression increased significantly (OR = 5.517, 95%CI = 2.434-12.504). Multinomial logistical regression analysis indicated that HIF-lα overexpression was an independent risk factor of AUR. As shown in Table 4, the OR of HIF-lα had been adjusted by age, IPSS as well as QoL score (OR = 0.154, 95%CI = 0.050-0.471). Above all, moderate-positive expression of HIF-lα in prostate intra-acinar was associated with the incidence rate of AUR.

**Table 4:** Multiple regression analysis of relationship between clinical variables and AUR incidence

	*P* Value	OR	95% C.I.
Age	0.245	1.062	0.960-1.175
IPSS Score	0.002	0.645	0.490-0.850
QoL Score	0.002	0.080	0.016-0.403
Wt^*^	0.086	0.960	0.916-1.006
HIF-lα	0.001	0.154	0.050-0.471

* Wt indicates the weight of intra-acinar of BPH

## DISCUSSION

In the present study, the expression of HIF-1α was evaluated by immunohistochemistry in tissues of fetal prostates, normal prostates, intra-acinar of BPH, peri-acinar of BPH, prostate cancers and sarcomas of prostate. With the clinical information of 95 BPH cases, HIF-lα expression in intra-acinar was found to be significantly correlated with the intra-acinar weight of hyperplastic prostate. Moreover, the incidence of AUR for HIF-lα moderate-positive patients increased significantly in this study, and HIF-lα is an independent risk factor for AUR.

Our data is in accord with the current opinion that prostatic hypoxia is responsible for the development of BPH [4-6]. Under normoxia, hydroxylated HIF-lα is recognized by the β-domain of von Hippel-Lindau tumour suppressor protein (pVHL) and is subsequently ubiquitylated by the Elongin BC/Cul2/pVHL ubiquitin-ligase complex [7]. The ubiquitylated HIF-lα could be degradated by the 26S proteasome.

However, under hypoxic conditions or in VHL^−/−^ cells, stabilized HIF-lα dimerizes with HIF-β and then bind to hypoxia-response elements (HREs), stimulating the expression of profuse hypoxia response genes including those encoding erythropoietin (EPO), vascular endothelial growth factor (VEGF), which stimulates erythropoiesis, angiogenesis, glycolysis, and invasion [13]. To date, little is known about the consequences of hypoxic conditions on human benign prostatic tissue as well as its clinical variables.

The prostate is an hormone sensitive organ which needs proper androgen receptor(AR) signals for normal growth [14]. Circulating androgen and the AR signaling, both epithelial and stromal AR, play critical roles in the pathogenesis of BPH, and that partially blockade of AR signaling, such as 5-α reductase inhibitors, decreases the BPH volume [15, 16]. *In vitro* studies demonstrated that dihydrotestosterone (DHT) activated the HIF-1 mediated gene expression, and hypoxia enhanced the AR-induced promoter activity of human PSA gene in prostate cancer cells [17]. HIF-lα might be activated by the AR signaling pathway and enhanced gene expression which is associated with proliferation during BPH. Several investigations suggest that aging disrupts the balance between proliferation and apoptosis of prostate cells which leads to BPH [18-20]. It's well established that HIF-lα has a protective effect against apoptosis [21]. Therefore, the expression of HIF-lα in BPH might cause resistance to apoptosis and preference for proliferation. An important theory for the mechanism of BPH is the re-awakening of embryo theory, presented by John McNeal, that the neo-formation of prostatic ductal-acinar tissue in the pathogenesis of BPH was due to the re-awakening of embryonic inductive activity by adult prostatic stroma [22]. We hypothesized that HIF-lα might mediate the development of BPH through re-awakening of embryo mechanism, since the expression of HIF-lα in FP was confirmed. Our data indicates that HIF-lα might play a significant role in the pathogenesis of BPH and the re-waking of prostatic stroma, in addition to its role in development of embryonic prostate. Moreover, growth factor from HIF-lα downstreams, including epidermal growth factor (EGF), transforming growth factor α(TGF-α), TGF-β, and basic fibroblast growth factor (bFGF), are involved as facilitators in the development of BPH [23]. View in toto, overexpression of HIF-lα is associated with BPH development. In addition, the tissue growth is dependent on its blood supply and thus induction of new blood vessels through angiogenesis is critical. The hyperplasia of intra-acinar of the prostate may be correlated with the hypoxic mircoenviroment, which stimulate angiogenesis through HIF-1α pathway [24]. Our clinical experience is another evidence for this theory that the bigger the BPH is, the harder it is for coagulation bleeding during the transurethral resection of the prostate(TURP). Further studies will be done about the relationship between HIF-1α expression and the bleeding during and post operation.

AUR is a severe symptom of patients with BPH, which defined as a sudden and painful inability to void voluntarily [25]. Progression of BPH leading to a mechanical obstruction of the bladder outlet, is the primary reason for AUR. It has been reported that men with AUR have higher mortality and morbidity rates [26]. There is a general consensus that men presenting with AUR had a high risk of death after operation and an increased risk of developing perioperative complications [27]. Prostatic inflammation has been reported to be an important risk factor in AUR etiology [28]. The expression of HIF-1α can be triggered by hypoxia, but also by pathological stress, such as inflammation and cancer [29]. And recent study showed that inhibition of inflammation via attenuating the expression of NF-kB and HIF-1α could prevent against experimental BPH. These evidence indicate that HIF-lα could be a bond between inflammation and AUR. The present study demonstrated that the expression of HIF-1α was significantly correlated with the incidence rate of AUR. Therefore, HIF-lα is a promising prognosis marker due to its association with prostate weight and the occurrence of AUR.

Inhibition of HIF-1α is a promising therapy, since its over expression correlated with poor prognosis in addition to BPH [30]. It has been reported that rapamycin could decrease levels of HIF-1α both under normoxia and hypoxia conditions [31]. The mechanism might be the blockade of mammalian target of rapamycin (mTOR)-dependent increase in HIF-1α and HIF-2α protein levels and VEGF upregulation. In addition, rapatar, a nanoformulation of rapamycin, has been demonstrated to decrease chemically-induced BPH in two rat models, without obvious side effects [32]. Moreover, increasing studies have demonstrated that rapamycin prolongs life span and prevents age-related diseases in mice [33-35]. Coincidentally, BPH is one of the most common age-related diseases and its pathogenesis involves changes of systematic hormone level and cellular metabolic pattern with the increase of age. By preventing age-related weight gain and decreasing rate of aging, rapamycin can also delay the occurrence of risk factors for BPH [34]. Therefore, HIF-1α could be the bond between mTOR and BPH, but further studies are needed to fully illustrate the mechanism of rapamycin treatment for BPH.

In conclusion, the expression of HIF-lα in prostates and its correlation with clinical variables were investigated. Our data demonstrated that HIF-lα expresses in all kinds of actively growing prostatic tissues, such as FP, intra-acinar of hyperplasia tissues of BPH and prostate malignant tumors. The expression of HIF-lα cannot be detected in the tissues of NP or peri-acinar of BPH. We also found that the levels of HIF-lα was significantly correlated with the weight of intra-acinar of prostate (*P* < 0.05). And patients with prostate weight larger that 72.45g were associated with HIF-lα moderate-positive expression. Moreover, HIF-lα is an independent risk factor for AUR, and the risk of AUR for HIF-lα moderate-positive patients increased significantly in this study, which could be a marker for the prediction of AUR.

## MATERIALS AND METHODS

### Ethics statement

All procedures were consistent with the National Institutes of Health Guide and approved by Institutional Board with patients’ written consent. This study was evaluated and approved by the Ethics Committee of Provincial Hospital Affiliated to Shandong University.

### Collection of specimens and patient enrollment

All the pathologically confirmed specimens were collected from patients between February 2012 and October 2014 in Provincial Hospital Affiliated to Shandong University, P.R. China. For control groups, 12 cases of the fetal prostate were collected from fresh male fetal corpse (induction delivery or miscarriage), Department of Obstetrics, with fetal age ranges from 20∼32 weeks; 10 cases of normal prostate specimens were collected from fresh male corpse, aged 20∼39 years, 30 years in average; 7 cases of prostate cancer specimens were obtained from the transition zone of radical prostatectomy specimens, with Gleason score ranged from 5∼9; 2 cases of sarcomas of prostate were collected from patients undergoing radical prostatectomy.

BPH patients eligible for this study included men aged 50 years or more. Diagnosis were confirmed by prostate-specific antigen, transrectal ultrasound, digital rectal examination, urine flow measurements, International Prostate Symptom Score (IPSS) and Quality of Life (QoL) scores. We excluded patients with prostate cancer, urinary tract infection, or a history of urologic surgery. 95 BPH specimens were collected from patients undergoing suprapubic prostatectomy, aged from 51∼85years, 67.8 years in average(mean IPSS socore = 26.45, QoL score = 4.86); 12 cases peri-acinar of BPH were collected from patients undergoing total cystectomy or prostatectomy.

Patients information included age, pathological type, and the weight(Wt) of the gross specimens. The prostate weight of all the adult patients ranges from 25.5g∼150.6g (68.5g in average). According to the classification standard of BPH from Rous(1985), all the specimens were divided into 3 Wt groups: group A (25g∼50g) 18 cases, group B(50g∼75g) 38cases, group C (﹥75g) 39 cases. Medical history was reviewed and recorded, including the incidence of acute urine retention(AUR).

### Immunohistochemistry of HIF-lα

All the prostate tissues were fixed in 4% formalin overnight and embedded in paraffin with standard techniques. 5 μm sections were cut (Leica-2135) and stained with Hematoxylin-Eosin (HE). The immunohistochemistry detection of HIF-lα was performed with the SABC kit (Boster Bioscience). Hydrogen peroxide was used to deactivate intrinsic peroxydase. Antigen retrieval was performed in a water bath using citrate-EDTA buffer (10mM citric acid, 2mM EDTA, 0.05% Tween 20, pH 6.2). Sections were incubated with diluted anti-HIF-lα antibody (1:60; Santa Cruz) or rabbit anti-VEGF antiboday (1:200; Abcam) overnight at 4°C. As negative controls, immunostaining was performed by incubating samples with PBS instead of primary antibody. Add biotin labeled secondary antibody to the slides followed by adding horseradish peroxidase (HRP) labeled streptavidin. After staining with DAB and counterstaining with hematoxylin, the slides were recorded using a digital camera (Leica-DM4000B).

### Evaluation of HIF-lα immunoreactivity

At least 100 epithelial cells within each area were evaluated at 400× magnificaction under microscope. Areas of prostatitis and prostatic stroma were not considered for evaluation. Cells were counted and averaged in five high power fields from each section. The immunohistochemical results for HIF-lα protein were classified into four grades as follows: (−) no staining or no difference in coloration from adjacent tissues; (+) nuclear staining in more than 10% of cells, colored straw yellow or yellow; (++) nuclear staining in 30%∼60% of cells, colored yellow or brownish yellow; (+++) nuclear staining in more than 60% of cells, with a strong colored intensity, brownish yellow or even brownish black. The positive control sections were provided by Boster Bioscience, and the negative control is the PBS incubated group.

### Statistical researching

Statistical analyses were performed with SPSS (Statistical Package for Social Sciences, Chicago, USA) software (Version 23). Differences of HIF-lα expression between intra-acinar or peri-acinar of BPH were analyzed by Chi-Squar methods. For comparison of BPH tissues and FP or tumors, Fisher exact probability test was applied. Means and standard error (SE) were used to describe continuous variables. In addition, ANOVA test, Chi-Squar test, receiver operating characteristic curve were used for investigating the association between HIF-lα expression in intra-acinar of BPH and the Wt of intra-acinar. Multinomial logistical regression was applied to explore risk effect of HIF-lα expression on the occurance of AUR. The conventional *P* < 0.05 was used to assess statistical significance.
